# Editorial: Family and school influences on individuals' early and later adjustment

**DOI:** 10.3389/fpsyg.2024.1437320

**Published:** 2024-06-14

**Authors:** Isabel Martínez, Iris M. Oliveira, Sergio Murgui, Feliciano H. Veiga

**Affiliations:** ^1^Department of Psychology, University of Castilla-La Mancha, Cuenca, Spain; ^2^Faculty of Philosophy and Social Sciences, Universidade Católica Portuguesa, Braga, Portugal; ^3^Department of Social Psychology, University of Valencia, Valencia, Spain; ^4^Instituto de Educação, Universidade de Lisboa, Lisboa, Portugal

**Keywords:** family, school, peer relationships, school adjustment, household chaos, parental warmth, childhood experiences

Family relationships have typically been addressed as the main basis for children's adjustment, which is consequently linked to individuals' later adjustment (Martínez et al., 2021). The family, as a primary group, is one of the main contexts involved in the socialization process. Family socialization and its effects on behavior, personality, or emotional adjustment have been a source of constant concern among psychologists and educators from diverse orientations and perspectives (Bales and Parsons, 2014). In most societies, the socialization of children is assumed to be a great responsibility, and the ways parents achieve it vary not only among cultures but also across families (Garcia et al., 2019). These differences are reflected in children's behaviors and their adaptation to societal challenges later in life as adults. Peers also play an important role in the socialization process. In addition to influencing the formation of values and moral development, the relationships established with peers have a clear influence on individual behavior (Martínez et al., 2022). In this context, the school constitutes the main place where relationships with peers are developed (Lam et al., 2014), in addition to other agents (e.g., teachers), thereby influencing one's behavior within and outside the school. Thus, the experiences at school, student engagement—both socially and academically—and the school performance itself will definitely influence the adjustment in adult life (Carvalho and Veiga, 2023).

The studies included in this Research Topic address different aspects of family and school relationships, illustrating their influence on personal adjustment. Five quantitative studies were published, showing results from three different continents: Asia (two studies from China; one from Korea), Europe (one study from Romania), and America (one study from the United States). The size of the samples ranges from 414 to 6,945, with *N* mostly being ~1,000 participants. The ages of the study subjects range from 5 to 6-year-old children to adolescents, covering the stages of preschool, elementary, and high school, with the majority (60%) focusing on preschool children. All studies present descriptive and non-experimental designs, analyzing mostly children and adolescents' self-responses. However, some studies also include questionnaires answered by parents and teachers, with a predominance for Likert-type scales, and one study uses a polytomous response survey. Most data analyses rely on statistical regression, with three studies focusing on mediating effects. One of the studies classified participants [using Landscape Reconstruction Algorithm (LRA)] and compared their scores with Multivariate analysis of covariance (MANCOVA). Hence, most analyses focus on the relationship between variables rather than on differences in independent constructs.

The five studies united in this Research Topic consider specific aspects of the family climate, such as parenting styles, parent-child interaction, or chaos at home. The school's climate is analyzed based on factors such as the closeness between teachers and students and the number of friends and peers' acceptance. The adjustment indicators considered include variables capturing the child's way of playing, self-efficacy, his/her approach to studying, academic performance, and perceived academic competence. Receptive language, social withdrawal behaviors in class, self-esteem, and satisfaction with life are other variables addressed.

Three of the studies are focused on the preschool stage. In the first focused on preschool theme, Zhan and Guo examine whether children's executive function and receptive language mediate the relationship between household chaos and social withdrawal. The results indicate that household chaos is indirectly associated with children's social withdrawal through the mediating roles of executive function and receptive language ability. Hence, household chaos constitutes a risk factor, but children's executive function and receptive language ability are protective factors against social withdrawal. The authors call for research on children's executive functioning and language abilities, acknowledging the importance of practices fostering these processes. In the second study focused on preschool age, Liu et al. associate parental warmth with children's approaches to learning, testing the mediation of children's self-efficacy perceived by parents and the moderation of teacher-child closeness. Findings suggest that parental warmth is positively associated with children's approaches to learning and that this association is mediated by self-efficacy and moderated by teacher-child closeness. The authors recommend future research on the antecedents of children's approaches to learning and call for practices prompting children's self-efficacy and approaches to learning. Finally, in the third study that analyzes preschool children, Lee identifies children's profiles according to individual, family, and preschool environmental factors, in addition to examining how such profiles are related to school adjustment, academic achievement, and executive function later in the first grade. Five profiles are identified, differing mostly on environmental rather than individual factors (“Good Social Competence by Good Educational Environment,” “Good Social Competence by Good Family Environment,” “Moderate,” “Poor Social Competence by Poor Educational Environment,” “Poor Social Competence by Poor Familial Environment”). In the first grade, the first two profiles demonstrate greater school adjustment, academic achievement, and executive function than the third profile. The authors claim the need for longitudinal studies and family-school collaboration to jointly promote children's adjustment.

Of the two remaining studies, the study by T´epordei et al. focuses on elementary school students, examining the role of peer relationships on the academic and affective processes. The results indicate that peer acceptance and the number of friends are positively associated with pre-adolescents' perceived academic competence, which in turn is positively associated with academic achievement and life satisfaction. The authors call for future longitudinal studies and school climates to feature supportive peer relationships. Finally, the study by Iverson et al., which uses a sample of high school students, associates adverse childhood experiences with perceived cognitive difficulties after controlling for the effects of poor mental health, depression, and suicidal ideation. These findings indicate that parental emotional and physical abuses are positively associated with cognitive difficulties. Among girls, sexual assault and sexual violence are also positively associated with cognitive difficulties. The authors advertise for the long-term risks of adverse childhood experiences and recommend therapies targeting youths' cognitive processes and emotional competencies.

Overall, the studies that comprise this Research Topic are aligned with a bioecological framework of human development, acknowledging person-in-context interactions and the pathways of adjustment from childhood through adolescence. Some studies cover family-school factors, thus illustrating the importance of addressing this mesosystem. Although the peer group is often less studied than the family or other school relationships, the importance of this social space is herein illustrated. Finally, the studies suggest that emotional processes and past experiences can impact one's adjustment. Hence, research on school success and adjustment needs to acknowledge the interplay between the cognitive and emotional aspects.

## Author contributions

IM: Writing – original draft, Writing – review & editing. IO: Writing – original draft, Writing – review & editing. SM: Writing – original draft, Writing – review & editing. FV: Writing – review & editing, Writing – original draft.
